# Isolation of Thymol from *Trachyspermum ammi* Fruits for Treatment of Diabetes and Diabetic Neuropathy in STZ-Induced Rats

**DOI:** 10.1155/2022/8263999

**Published:** 2022-04-28

**Authors:** Neetu Sachan, Nikita Saraswat, Phool Chandra, Mohammad Khalid, Atul Kabra

**Affiliations:** ^1^School of Pharmaceutical Sciences, Faculty of Pharmacy, IFTM University, Lodhipur Rajput, Delhi Road, NH-24, Moradabad (UP)-244 102, India; ^2^Institute of Pharmacy, Pranveer Singh Institute of Technology, Kanpur, Kanpur-Agra-Delhi National Highway-2, Bhauti, Kanpur (UP)-209305, India; ^3^Dr. D. Y Patil College of Pharmacy, Akurdi, Pune, Maharashtra-411044, India; ^4^Department of Pharmacognosy, College of Pharmacy, Prince Sattam Bin Abdulaziz University, Al-Kharj 11942, Saudi Arabia; ^5^University Institute of Pharma Sciences, Chandigarh University, Gharuan, Mohali, Punjab, India

## Abstract

Terpenoids and phenols from *Trachyspermum ammi* (*T. ammi*) have reported some pharmacological actions. The objective of the work was to isolate the active constituent, its identification by spectroscopic techniques, and evaluation of the antidiabetic and neuroprotective activity from *T. ammi* on STZ Wistar rats. The dried fruits of *T ammi* were kept in a hydrodistillation apparatus to collect essential oil. The isolated fraction went through TLC, UV, FTIR, HPLC, HRMS, C^13^, and ^1^H NMR for characterization. Two dosage concentrations from the isolated compound were prepared as 10 and 20 mg/kg for treatment groups. The groups were tested for thermal and mechanical hyperalgesia, writhing, grip strength, spontaneous locomotor test, neuromuscular coordination tests, and histopathological and lipid profile analysis. Diabetes was induced by streptozotocin (45 mg/kg i.p.) and 12 weeks of treatment-induced diabetic neuropathy in Wistar rats. Biomarkers were evaluated to understand the neuropathic protection of thymol on STZ-treated Wistar rats. The biomarker studies (SOD, NO, LPO, Na^+^K^+^ATPase, and TNF-*α*) further confirmed thymol's diabetic neuropathy protective action. This study suggests that isolated compound thymol was antidiabetic and neuroprotective as it has shown controlled glucose levels defensive nerve damage in STZ Wistar rats. *P* < 0.05 level of significance was observed in the levels of endogenous biomarkers, fasting blood glucose levels, actophotometer response, and response latency in treated groups compared to the diabetic group, whereas *P* < 0.001 level of significance during lipid profile levels, thermal algesia, and neuromuscular comparison tests was noted in treated groups compared to the diabetic group.

## 1. Introduction

Patients suffering from diabetes mellitus (DM) observe a severe condition of peripheral nerve dysfunction called diabetic neuropathy (DN) [1, 2]. In India, the prevalence is higher (4.3%) compared to the western countries where 1%–2% population of DM faces these conditions. This could be due to the probability that Asian Indians are prone to the condition of insulin resistance [3–5]. Around 2/3rd of the diabetic population suffers from clinical or subclinical neuropathic conditions, where approximately 10% of the people with diabetes face persistent pain. Diabetic neuropathy has a common feature of spontaneous pain, intractable or stimulus-induced pain [6]. There are many categories of DN where diabetic polyneuropathy (DPN) is a condition that prevails for a lifetime and is a major cause of nerve injury, foot ulceration, gait disturbance, and amputation [7–11]. The central control observed for DN is preventive management, as the check on the glycemic control helps prevent neuropathic complications [12].

The negative symptom observed in patients suffering from DN is a decrease in sensation-induced numbness, whereas the positive symptoms observed are aching, prickling, and burning sensations [13]. Mechanisms suggest that the tiny and unmyelinated nerve fibres are responsible for conveying sensations like temperature, touch, and pain, while the long white fibres get sensations of joint position and vibratory senses. Most of the patients report mild discomfort but around 25% report painful neuropathic conditions in diabetes. Pain in diabetic neuropathy often worsens at night [1, 14–16].

Diabetes has gradually become the most prominent problem in the global healthcare issue of the 21st century. The population of diabetics is predicted to double between the years 2000 to 2030 by reaching to level of 366 million people [17, 18]. This metabolic disorder shows prominence of hyperglycemia and includes defects of insulin productions or insulin secretion or both. Cases of chronic hyperglycemia in diabetes lead to dysfunctions and damages in the kidney, nerves, eyes, heart, and blood vessels. Prominent symptoms of hyperglycemia include weight loss, polydipsia, polyuria, blurred vision, and sometimes polyphagia. Susceptibility to infections and impairment of growth are chronic effects of prolonged hyperglycemia. In contrast, the acute fatal consequences of diabetes are nonketotic hyperosmolar syndrome and hyperglycemia with ketoacidosis [19, 20].

Prime causes of Type 2DM are genetics and lifestyle factors [21]. Advancing age is also a risk factor of T2DM, but the increasing incidences of obesity in childhood have resulted in prominent cases of T2DM in adolescents, children, and teenagers which is a serious concern and an emerging public health epidemic [22].

*T. ammi* is commonly known as “Ajwain” and is found in major parts of India, dominantly in Rajasthan and Gujarat regions. The plant belongs to the family Umbelliferae and also is called Omum, Ajowan, in Sanskrit [23, 24], Agyptischer in German, Kammun or Al-Yunan in Arabic, Hounastan in Armenian, and Xi Ye Cao Guo Qin in Dutch. This plant is native to Indian region, Pakistan, Iraq, and Afghanistan but also grows in regions near Mediterranean Sea, Egypt, and southwest Asia. The herb possesses many medicinal values and is used traditionally for curing conditions like atonic dyspepsia, abdominal tumors, lack of appetite, flatulence, bronchial problems, abdominal pains, diarrhea, piles, asthma, and amenorrhoea. Many pieces of research have proved the plant to be antinociceptive, hypolipidemic, antimicrobial, abortifacient, nematicidal, antioxidant, antifungal, antispasmodic, anti-hypertensive, antilithiasis, diuretic, antitussive, antifilarial, abortifacient, hypolipidemic, and cytotoxic and having bronchodilating actions [25].

Currently, a huge inclination to the herbal sources is noted in the case of many treatments. Since the ancient era, humans have depended on natural sources as remedies for diseased conditions. In India, Ayurvedic remedies are used as treatments for ages. Due to reported adverse reactions in synthetic medications and temporary relief, a huge population has drifted to natural and herbal sources for curing ailments. There has been a lot of reaches where herbs and their extracts have been proved useful in the treatment of diseases [26–29].

Based on the literature review, we found no study on diabetes and diabetic neuropathy of thymol isolated from dried fruits of *T. ammi*. Therefore, we planned to isolate thymol (an active constituent) from the dried fruits of *Trachyspermum ammi* and evaluate its effects on diabetes and diabetic neuropathy by experimentally inducing it on animals.

## 2. Material and Methods

### 2.1. Drugs and Chemicals

Streptozotocin was acquired from Sigma-Aldrich India, while Sanofi India Ltd. provided glibenclamide for a research work. The chemicals were of analytical grade obtained from the laboratory facility of the institute.

### 2.2. Instruments

UV-1700 PharmaSpec on SHIMADZU, FTIR–Spectrum Two on PerkinElmer, and HPLC on LC-2010CHT by Shimadzu were used from PSIT, Kanpur laboratory facility for performing UV and FTIR spectroscopy, while C^13^ NMR and ^1^H NMR were performed by Indian Institute of Technology, Kanpur laboratory facility, where DMSO−D6 was used as solvent. The HRMS was done by SAIF CDRI, Lucknow, where ethanol was used as a solvent. A glucometer (Accu-chek®) was used to estimate blood glucose levels and a semiauto analyzer (Remi Industries Ltd., India) was used for biochemical analysis.

### 2.3. Test Animal

**Figure 1:**
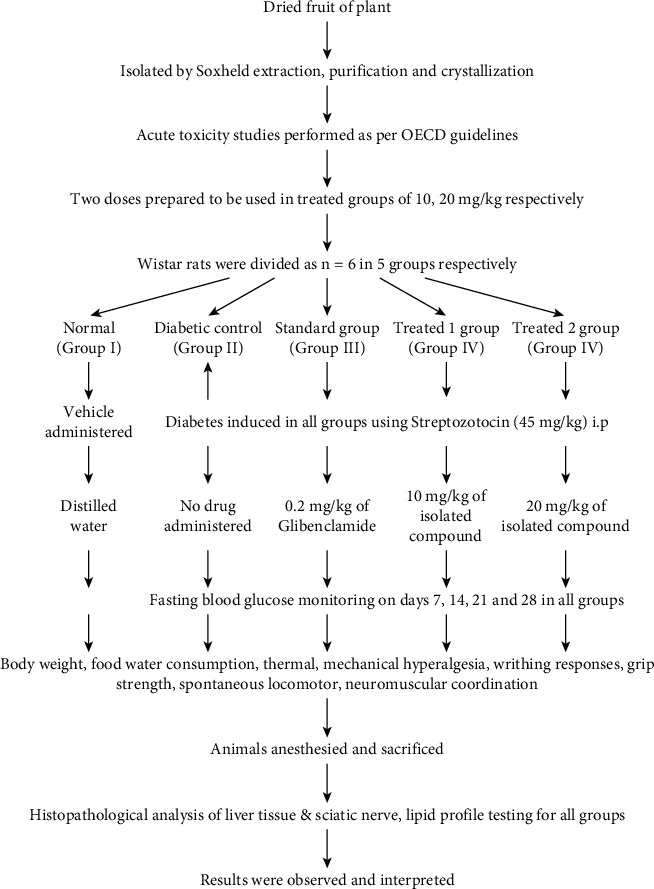
Experimental plan.

Adult rats weighing (180-220 g) of both genders were obtained from the animal house of Institute of Pharmacy, PSIT, Kanpur. Animals were kept in cages (polyacrylic) which were spacious and large. An ambient room temperature was maintained with 12 h light/12 h dark cycle. Purified water and standard pellet diet ad libitum were made available to experimental rats [30]. This study was approved by IAEC, and the experiment was performed in accordance to CPCSEA (Ref No. 1273/PO/Re/S/09/CPCSEA). The experimentation design chosen is mentioned in Table 1, and the process is explained in Figure 1.

### 2.4. Collection and Identification of Plant Material

**Figure 2:**
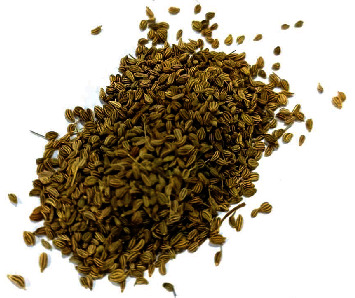
Fruit of *Trachyspermum ammi* used for isolation of active compound.

**Figure 3:**
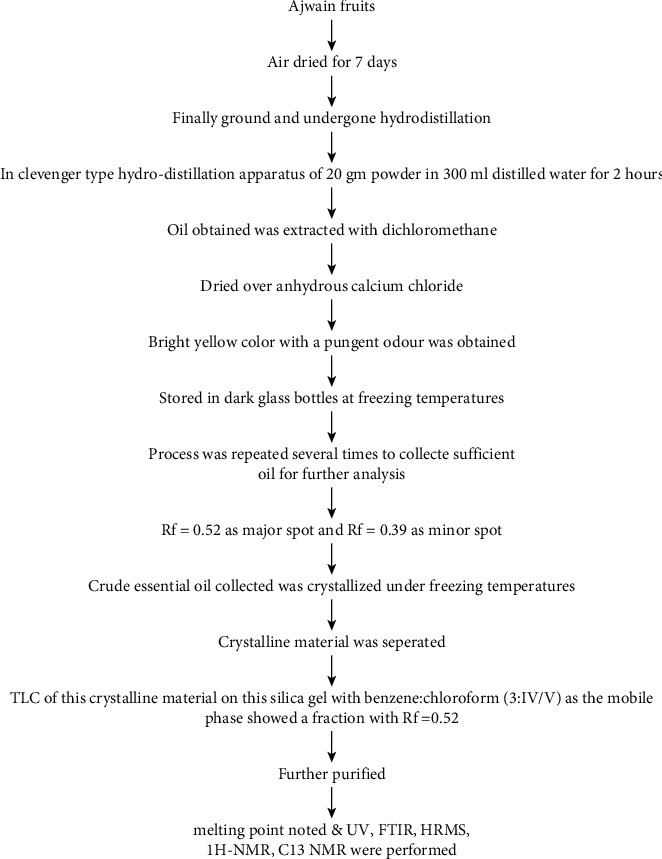
Isolation of thymol from the fruit of *Trachyspermum ammi.*

The fruit of *T ammi* was obtained from the local market in Kanpur, Uttar Pradesh, India (Figure 2). They were collected in November 2018. The powdered fruit underwent hydrodistillation and was purified to obtain an isolated compound (Figure 3).

### 2.5. Identification of Compound by TLC

The TLC of the crystalline material obtained after hydrodistillation and purification showed an *R*_*f*_ value of 0.52. The system of TLC used had silica gel (stationary phase) and benzene : chloroform (3 : 1 *V*/*V*) as the mobile phase.

### 2.6. Identification of Compound by ^1^H NMR, C 13 NMR, HPLC, HRMS, UV, and FTIR Analysis

**Figure 4:**
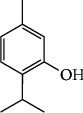
Structure of thymol.

The crystalline material obtained from the Crude Essential oil collected was taken for UV, HPLC, and FTIR spectroscopy studies using ethanol as a solvent. These studies were carried out in the Department Pharmacy of PSIT (Kanpur), and the results obtained are linked. The HRMS for the estimation of compound molecular weight was performed by SAIF, CDRI, Lucknow, using ethanol as a solvent. The ^1^H NMR and C^13^ NMR were performed by the Indian Institute of Technology, Kanpur, where DMSO−D6 was used as a solvent. The melting point was registered as 50°C using a melting point apparatus. On analysing the TLC results and all spectroscopies, the interpretation of the compound was found to be thymol (Figure 4).

### 2.7. Acute Toxicity Study

**Figure 5:**
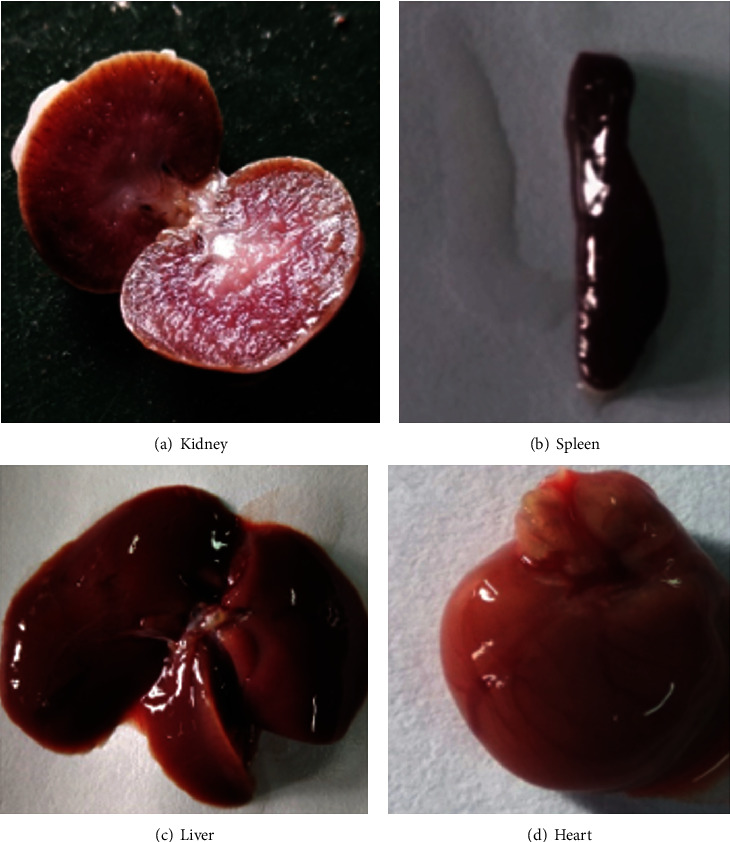
Observation of visible changes on organs postacute toxicity studies.

The ethanolic extract of *Trachyspermum ammi* was checked up to a dose of 3200 mg/kg orally, and it was hence confirmed that the lethal dose was beyond 3200 mg/kg (b.wt) [31]. The acute toxicity experiments were done in accordance with guideline no. 425 of the OECD. The treated rats were given isolated compound doses of 5, 50, 300, and 2000 mg/kg, respectively. The dose chosen for the activity was 10 and 20 mg/kg (b. wt.) of compound isolated. Rats were then checked for their behaviour like irritability, alertness, restlessness, and fearfulness. They were also checked for neurological abnormalities like pain response, touch response, gait and spontaneous activity, and autonomic reflex-like urination as well as for defecation for 24 hours. 14 days from the administration of dose, the animals were observed for any case of mortality, and on the 14th day, they were sacrificed to isolate organs to note any visible morphological changes or signs of toxicity (Figure 5) [32–34].

### 2.8. Induction of Diabetes Mellitus

An intraperitoneal route was used for administration of streptozotocin (Sigma-Aldrich) at a dose of 45 mg/kg post overnight fasting of at least 12 hours to induce hyperglycemia. The hyperglycemia was confirmed post 72 hours of STZ injection and was marked by increased blood glucose levels. After the induction of diabetes, the animals were randomized as per their body weight and glucose levels thus having 4 groups according to protocol. A control group was established where diabetes was not induced. Estimation of fasting blood glucose on 7th, 14th, 21st, and 28th days was done via a glucometer from Accu-chek® [35].

### 2.9. Induction of Diabetic Neuropathy

Diabetes was induced, and 12 weeks after the experimentation, its effects were evaluated on the nerves to find out the possibilities of generation of diabetic neuropathy. The neuropathic development was assessed with the help of various parameters like locomotor tests and examination of the nerve tissue morphologically and microscopically on weeks 2, 4, 8, and 12 in every group.

#### 2.9.1. Estimation of Fasting Blood Glucose

In each group, rats were fasted one day prior to testing their glucose levels on days 7, 14, 21, and 28 of experimentation. Glucose levels were obtained by collecting small amount of blood from tail and tested via a diagnostic kit [35].

#### 2.9.2. Body Weight, Food, and Water Consumed

The body weight for each animal, consumption of food, and water were monitored during the experiment, recorded, and compared [36].

#### 2.9.3. Estimation of Lipid Profile

Serum was separated by the help of centrifugation process by using centrifuge at a speed of 15000 rpm for 10 min—Remi Industries Ltd. (Mumbai, India). The isolated serum was analysed for TC, LDL, HDL, and triglyceride analysis using a semiautoanalyser (Span Diagnostics Ltd., India). The lipid profile estimations were performed at the end of experimentation, and the blood was collected from the retroorbital method.

### 2.10. Behavioural Studies

#### 2.10.1. Thermal Hyperalgesia

Animals were kept on the analgesiometer (hot plate device) manufactured by Columbus instruments. 55 ± 1°C was set as the device temperature for the experiment. Initially, the symptoms like jumping or licking of paws were recorded and the reaction time in seconds was noted. Cut-off time for the investigation was 10 seconds to prevent any damages to rat paws. Eddy's hot plate method was used for testing the hyperalgesia on weeks 2, 4, 8, and 12 in all animals [37].

#### 2.10.2. Writhing Responses

Assessment of neuropathic pain was done by recording writhing responses in animals. 1% *v*/*v* acetic acid (in distilled water) was administered to rats in a volume of 0.1 ml/10 g body weight to stimulate writhing responses. All episodes of stretching of back, elongation of body, and limb extensions were noted and counted in all animals [38].

#### 2.10.3. Cold Hyperalgesia

The acetone drop test was conducted to evaluate the cold sensitivity of the rats. Animals from every group were kept in separate mesh cages for acclimatization. Fresh drops of 50 *μ*l acetone were then applied on the midplantar surface of paws gently. The stimulation of cold was created around 2-5 sec of application, and then the responses like mild paw withdrawal, shaking, rubbing, or licking of paw were noted. These reactions were noted as nociceptive responses, no responses (antinociceptive effects), or delayed responses (delayed nociception) in terms of minutes on both paws at intervals of 5 minutes; hence, the mean response was noted [39].

#### 2.10.4. Evaluation of Mechanical Hyperalgesia

A pinprick test was performed on rats (hind paw) where the paw was pricked with a 900 C bent gauge needle but no piercing was done. Reflexes were recorded for paw withdrawal postgentle prick, and responses were recorded in seconds. The cut-off time was 20 sec. Results were noted on weeks 2, 4, 8, and 12 of experimentation [40].

#### 2.10.5. Grip Strength

The tests were performed to determine the neuromuscular strength of animals by dangling them on forelimbs with metal wire held tight on the poles. Duration of falling was recorded before falling on the surface hence estimating their muscle strength. Time was noted, and the interpretations were made on the basis of time of fall (as a week or damaged muscles will lead to less time of fall) [41].

#### 2.10.6. Spontaneous Locomotor Testing

This test was performed by introspecting the animal's behaviour and activity in an actophotometer. Animals were kept in a 30 × 30 × 30 cm actophotometer with photocells and a digital counter to record the interruptions in the beam during activity recording [42].

#### 2.10.7. Estimation of Neuromuscular Coordination

A motor coordination test was performed using a rotarod apparatus where rotarod was operating on 25 rpm speed. Cut-off time was 5 minutes, and fall-off time was recorded for all animals during the test. The low fall of time reciprocates to damaged nerve and muscle [43].

### 2.11. Histopathological Study

**Figure 6:**
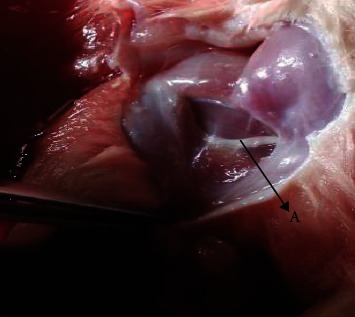
Isolation of sciatic nerve from the thigh region near lower limb of Wistar rat. (a) Sciatic nerve as observed in the rat was isolated from animals of all groups, freeze overnight in formalin, and sections were taken to compare the degenerations in respective groups.

Tissues were isolated from the liver and sciatic nerves of rats at the termination of experiment. On week 12 of the study, the sciatic nerve from the lower limb's thigh region and the liver from the study animal were isolated, washed, and then stored (Figure 6). The nerve and liver were cleansed and stored in 4% formalin solution with pH 6.9. They were kept at freezing temperatures dipped in 24 hours, and then, the histopathological analysis was carried out. The samples were precisely cut and isolated and then stained with eosin and hematoxylin [44, 45].

### 2.12. Biochemical Estimations

#### 2.12.1. Preparation of Homogenate of Sciatic Nerve

At the termination of the procedure, animals were exposed to a high dose of anesthesia to isolate the sciatic nerve. Homogenate of tissue was prepared using a buffer solution (0.1 M Tris–HCl) with a pH of 7.4. The supernatant fluid of the homogenate was used for the estimation of membrane-bound inorganic phosphate, SOD (superoxide dismutase), lipid peroxidation (MDA content-LPO), TNF-*α*, and NO content (nitric oxide) [46, 47].

#### 2.12.2. Evaluation of TNF-*α*

Quantification of TNF-*α* was done using a Thermo Scientific immunoassay kit for estimation of TNF-*α* levels in rat solid-phase ELISA for a duration of 4.5 h. A microplate was precoated with a monoclonal antibody, and 50 *μ*l buffer was added to each well plate.

Samples from homogenate obtained from sciatic nerve of each group were incubated at RT for 1 hour. Immobilized antibodies will bind to TNF-*α* present in the well.

Washing of sample was done followed by pouring of biotinylated antibody reagents into all wells, respectively, and was then incubated at RT for 1 hour. It was further followed by washing. Then, 100 *μ*l of streptavidin-HPR reagent was added in each well. Antibody-enzyme was then added to remove any traces of unbound antibody. 100 *μ*l of 3,3′,5,5′-tetramethylbenzidine (TMB)—a substrate solution—was poured which changed the color of product from blue to yellow. The color intensity was noted at 550 nm [46, 47].

### 2.13. Statistical Analysis

Experiments were done by grouping animals as 6 in each group. Quantitative data was tabulated via ANOVA (one-way) method, and post hoc analysis of data was done via Tukey-Kramer's test.

## 3. Results

### 3.1. Isolation

During the characterization of the isolated compound, it was analysed that UV *λ*max = 272.40; HPLC: retention time 9.94; HRMS-151– Molecular Ion Peak, 108 – Fragment Ion Peak; IR (KBr, cm -1): 1610 (C=C str., Aromatic ring), 1422 (O-H ben.), 1236.53 (C-O str.), 3227.02 (O-H str, Phenol), 2955.48 (C-H str). 1 H NMR (500 MHz, Chloroform) *δ* 4.5591 – 6.1085 (m, 3H, Ar-H), 12.32 – 3.36 (m, 4H), 7.02 – 3.36 (m, 3H), 4.0392 (s, 1H, -OH), 3.1482-3.4128 (m, 1H), 1.7083 (s, 3H), 1.7981-1.8462 (d, 6H); 13 C NMR (125 MHz, DMSO) *δ* 154.61, 135.53, 131.65, 125.94, 120.09, 116.01, 26.55, 22.84, 20.90. Its FTIR, C 13 NMR, 1 H NMR was compared with standards, and the compound was identified as 2-isopropyl-5-methylphenol–Thymol [48, 49].

### 3.2. Acute Toxicity

14 days from the dose of administration, the animals were observed for any case of mortality. On the 14th day, they were sacrificed to isolate organs to note any visible morphological changes or signs of toxicity. The rats from all groups were checked for neurological abnormalities and autonomic reflexes. No exceptions were reported, and all results were normal. The organs isolated were observed for any morphological changes, and no changes were found. The results are recorded in Figure 5.

### 3.3. Fasting Blood Glucose

Glucose levels rise uncontrollably in diabetic groups which were noticed as 308.27 ± 0.37 in Group II animals (Table 2). The levels of glucose were measured in all groups where it was found that Groups IV and V have shown 129.01 ± 3.45 and 117.12 ± 1.35 mg/dl concentrations, respectively, which shows improvement in glucose levels when glucose levels were measured in all groups where it was found that Groups IV and V have shown 129.01 ± 3.45 and 117.12 ± 1.35 mg/dl concentrations, respectively, which shows improvement in glucose levels compared with the diabetic control group on day 28 on the experiment. Group III has shown demonstrated improvement of glucose levels with 121.18 ± 3.23 mg/dl concentration on day 28.

### 3.4. Body Weight, Food, and Water Consumption

Bodyweight of Group IV and Group V was 201.34 ± 1.38 g and 211.23 ± 1.42 g at conclusion of the experiment, which is higher when compared against diabetic control (132.36 ± 3.26) and also efficient when compared with Group III (199.2 ± 1.12 g), while food intake was 22.47 and 21.02 g/day which is less than the diabetic control group (27.26 ± 0.31 g/day). The glibenclamide group (Group III) has shown efficient results with 21.14 ± 2.62 g/day food intake.

On recording the water intake, it was found that Group IV and Group V consumed 17.42 ± 1.35 and 16.13 ± 0.59 ml/day, which is less than the diabetes group, while Group III has shown 21.17 ± 2.35 ml/day of average water intake (Tables 3 and 4).

### 3.5. Lipid Profile

Groups IV and V have shown 85.02 ± 1.91 mg/dl and 74.59 ± 0.24 mg/dl levels of TC; 72.26 ± 2.43 mg/dl and 65.15 ± 1.28 mg/dl levels of triglyceride; 45.26 ± 2.36 mg/dl and 37.24 ± 2.29 mg/dl of LDL levels; 21.13 ± 1.27 mg/dl and 14.01 ± 0.13 mg/dl of VLDL levels; and 20.35 ± 2.26 mg/dl and 28.03 ± 0.29 mg/dl of HDL levels which are improved values compared with the diabetic group. The standard group has shown improvements in lipid profile with TC level as 77.23 ± 2.36 mg/dl, triglyceride as 65.48 ± 1.49 mg/dl, LDL levels as 37.16 ± 0.25 mg/dl, HDL levels as 29.8 ± 2.46 mg/dl, and VLDL levels as 15.12 ± 1.38 mg/dl which have shown improvements in comparison to the diabetic group [Table 5].

### 3.6. Behavioural Studies

#### 3.6.1. Estimation of Thermal Hyperalgesia

In Eddy's hot plate experiment, the response time recorded was 6.08 ± 1.41 s and 5.93 ± 0.24 s for Group IV and Group V, respectively, as recorded on the 12^th^ week. The results justify the healing of the nerves compared with Group II, which has recorded a higher (9.85 ± 1.86 s) response time due to potential damage to the nerves due to diabetic neuropathy. Group III has shown 5.91 ± 1.74 s as response time, which improves results (Table 6).

#### 3.6.2. Writhing Responses

Writhing responses have recorded values of 8.28 ± 1.44 and 8.24 ± 0.17 (number of reactions) on week 12, which have shown improved latency when compared against the diabetic control group (Group 2), where reported latencies on week 12 are high due to potential damage to the nerves. Group III responses have also improved (8.94 ± 1.35) [Table 7].

#### 3.6.3. Cold Hyperalgesia

When the acetone drop test results were compared from Group IV and Group V against diabetic control on 12^th^ week, the values obtained were 5.93 ± 1.38 and 5.15 ± 1.27 (sec). Here, an improvement in the latencies of the treated groups was noted compared to the diabetic group. Group III results were recorded as 6.11 ± 2.12 which had improvement compared to the diabetic control group (Table 8).

#### 3.6.4. Mechanical Hyperalgesia: Pinprick Test

On analysing the results of Group IV and V with Group II, readings of 4.89 ± 1.25 s and 4.51 ± 0.21 s have been noted on week 12 of experimentation. Though the changes have been quite significant throughout the experiment, the results on week 12 have shown improved results in the treated groups. The standard drug group has recorded a response of 4.74 ± 2.72 s which shows improvement (Table 9).

#### 3.6.5. Grip Strength

The animals in the diabetic group have lost their grip strength due to prolonged diabetes; hence, the diabetic group on week 12 shows poor results. In comparison, significant values of 24.35 ± 1.16 and 32.16 ± 1.62 (sec) were recorded for Groups V and V, respectively. Group III has recorded 28.21 ± 2.13 (sec) as grip strength. All treatment groups show improvement in the mechanical strength of animals (Table 10).

#### 3.6.6. Spontaneous Locomotor (Exploratory) Test

Significant readings of 93.25 ± 0.42, 90.33 ± 2.66, and 100.24 ± 2.55 (counts) were obtained on week 12 of experimentation in Groups III, IV, and V. The readings are very similar to normal groups and significantly different from the diabetic control; hence, they show an improvement in the spontaneous locomotor activity due to healing effect of drug (Table 11).

#### 3.6.7. Neuromuscular Coordination Testing

Motor coordination readings (rotarod test) for Groups III, IV, and V were recorded to be 109.13 ± 0.28, 100.25 ± 4.82, and 102.18 ± 0.31 which are significantly different compared to Group II on the 12th week of the experiment (Table 12).

### 3.7. The Histopathological Analysis

**Figure 7:**
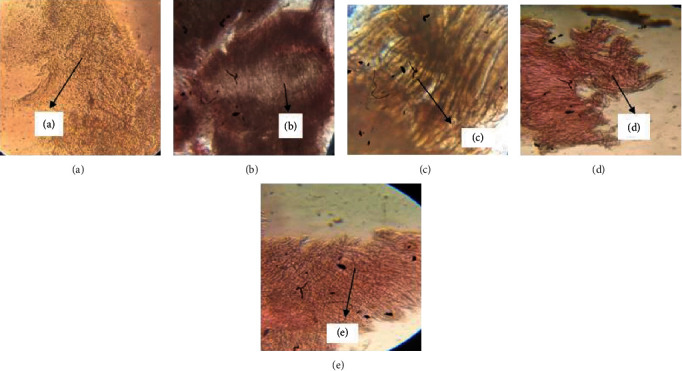
Analysis of longitudinal vasa nervorum in the sciatic nerves of Wistar rats on the 12^th^ week. Samples were stained and analysed using a microscope. Group II has shown signs of swelling in the neurons. Effects of thymol isolated from fruits of Trachyspermum *ammi* were noted on the treatment groups postdosing for 12 weeks. (a) Normal nerve tissue which is not showing any signs of inflammation or nerve degeneration, damage (control group); (b) untreated groups have shown prominent signs of inflammation and damage and lipid degenerated axons showing focal peripheral axonal loss (diabetic group); (c) mild focal axonal loss shows signs of recovery from the damage induced by prolonged diabetes hence signifying the effects of standard drug on the nerves; (d) mild focal axonal loss in peripheral region showing healing action of isolated compound post regularly dosing for 12 weeks; (e) minimal axonal degeneration noted in treated group II where 20 mg/kg of isolated compound dosing was performed, hence showing the strong healing and neuroprotective action of drug on the sciatic nerve tissue.

**Figure 8:**
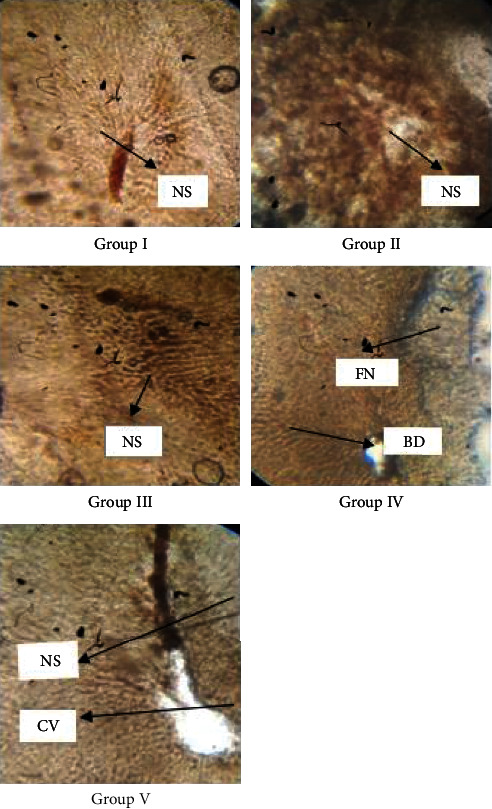
Histopathological analysis of the liver tissues which were obtained post 12 weeks of experiment (experimentally induced diabetic neuropathy) from all animal groups. Images represent the control group, diabetic control, standard, and treated groups. Samples were stained and show results as follows: (a) hepatic cells were observed as normal, and narrow sinusoids were clearly visible. (b) Fatty necrosis and ballooning degradation were noted in this image which represents disruptive cells in the liver. (c) Narrow sinusoids were observed in the slides, and comparatively healthy tissue was noted. (d) Normal hepatic cells with narrow sinusoids. (e) Normal hepatic cells with narrow sinusoids were observed. CV: central vein; NS: narrow sinusoids; FN: fatty necrosis; BD: ballooning degradation.

The histopathological analysis has shown normal results in treated groups, as shown in Figures 7 and 8. Little or no degradation was noted in sciatic nerve tissues similarly; no necrosis was noted in liver tissues of Groups IV and V. All results have shown significance against the diabetic control rats.

### 3.8. Biochemical Parameters

#### 3.8.1. Superoxide Dismutase Effect

The results have shown significant, dose-dependent results with isolated doses of thymol. A significant reduction in the value of superoxide dismutase was found (5.32 ± 1.13 U/mg of protein) in the homogenate of the sciatic nerve obtained from diabetic rats (Group II) compared with the normal control group (Group I), i.e., 24.03 ± 1.42 U/mg of protein. Rats from treated groups have shown rise in SOD levels (21.11 ± 1.23 and 23.24 ± 1.22 U/mg of protein) as shown in Table 13.

#### 3.8.2. The Effect of Thymol-Treated Groups on Nitrosative Stress

In the sciatic nerve, the neural nitrite levels from the diabetic groups were significantly increased to 303.11 ± 2.11 *μ*g/ml (*P* < 0.05) in comparison to the normal group recording values of 104.03 ± 1.36 (*P* < 0.05). Treated groups have shown values of 158.09 ± 2.12 and 129.22 ± 2.012 which are lowered significantly compared to the diabetic group rats (Table 13).

#### 3.8.3. Lipid Peroxide Profile Evaluation

Neutral Lipid Peroxidase (LPO) levels were recorded in all groups at the end of 12 weeks of study. The diabetic group has shown a significant increase (*P* < 0.05) in LPO levels. The value recorded for Group II was 9.11 ± 1.13 nM/mg of protein compared with the levels from the normal group (2.34 ± 1.13 nM/mg of protein). The treated groups recorded values as 4.13 ± 1.23 and 2.71 ± 1.31 nM/mg of protein (*P* < 0.05) (Table 13).

#### 3.8.4. Effect in Membrane-Bound Inorganic Phosphate

Na^+^K^+^ATPase level (membrane-bound inorganic phosphate) after 12 weeks of study was found to be 2.34 ± 0.17 *μ*mol/mg of protein with *P* < 0.05 in rats from the diabetic group which is low in comparison to normal group results (10.11 ± 1.23). The treated group rats have shown values of 7.31 ± 2.03 and 9.34 ± 1.34 *μ*mol/mg of protein which is significantly different from the diabetic group (Table 13).

#### 3.8.5. Effect in the TNF-*α*

A significant increase in TNF-*α* from sciatic nerves was noted in STZ diabetic groups (160.01 ± 1.21 pg/ml) when compared to the control group (51.35 ± 1.13 pg/ml). Treated groups have shown improved results with values of 90.21 ± 1.13 and 67.25 ± 0.21 pg/ml compared to the diseased groups (Table 13). Improvement in motor coordination was noted in treated group SOD; Na^+^K^+^ATPase values of treated groups receiving 10 and 20 mg/kg of thymol have shown a significant increase in the levels by *P* < 0.05 compared with those of the diabetic group. NO, LPO, and TNF-*α* values have a significant decrease in the levels by *P* < 0.05 compared to the diabetic group.

## 4. Discussion

A large part of the world's population suffers from diabetes whose major complications include hyperglycemia and diabetic neuropathy. Inflammation and hyperglycemia are the two events that alter gene expressions, thus affecting the cellular proteins, hence leading to progressive changes like diabetic complications and pathological changes. With the current growth status, diabetic neuropathy will be the prime cause of terminal stage renal diseases globally with intolerable consequences and costs for healthcare in developed countries [50, 51].

In the current treatment scenario, antidepressant drugs have been used in the treatment and regulation of neuropathic pain, which leads to the maintenance of sustained levels of neurotransmitters (norepinephrine and serotonin). As these hormones have shown a reduction in the central nervous system's pain pathways, they have shown good results in regulation and sensitivity to pain [52, 53].

**Figure 9:**
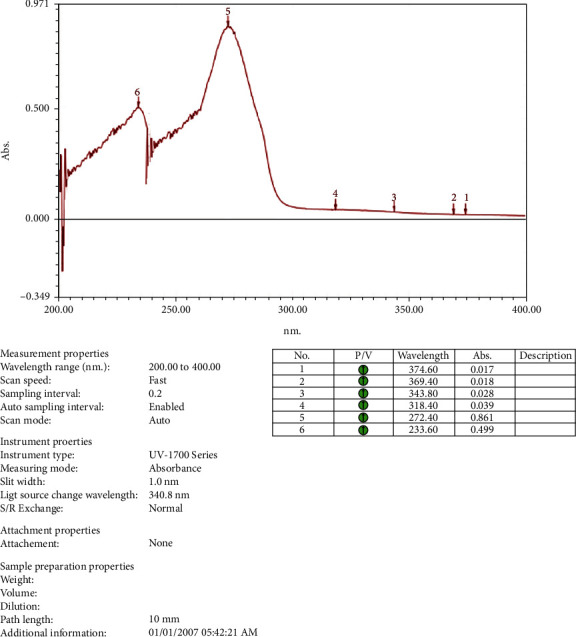
UV spectroscopy of the isolated compound.

**Figure 10:**
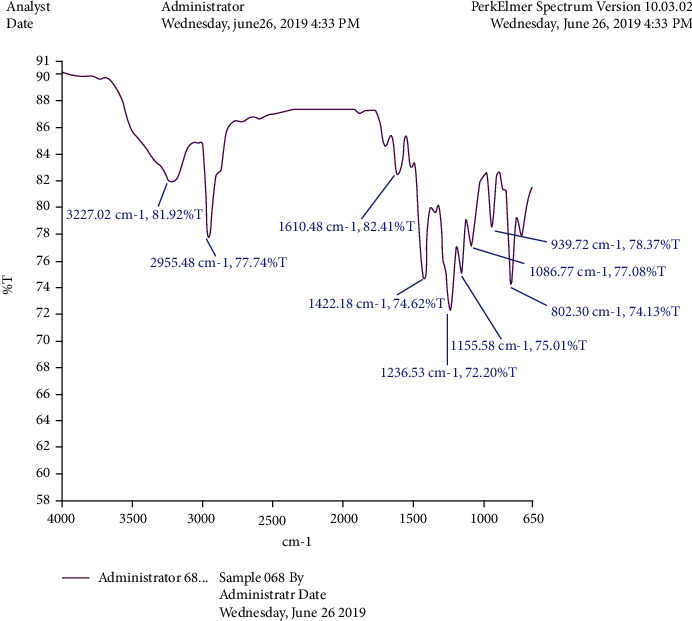
FTIR spectroscopy of the isolated compound.

**Figure 11:**
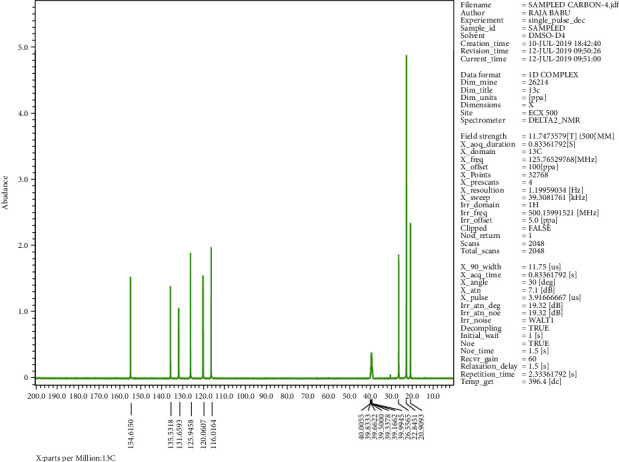
C^13^ NMR spectroscopy of the isolated compound.

**Figure 12:**
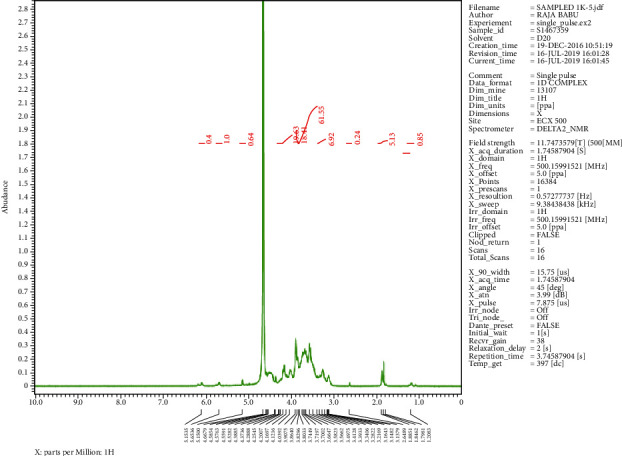
^1^H NMR spectroscopy of the isolated compound.

**Figure 13:**
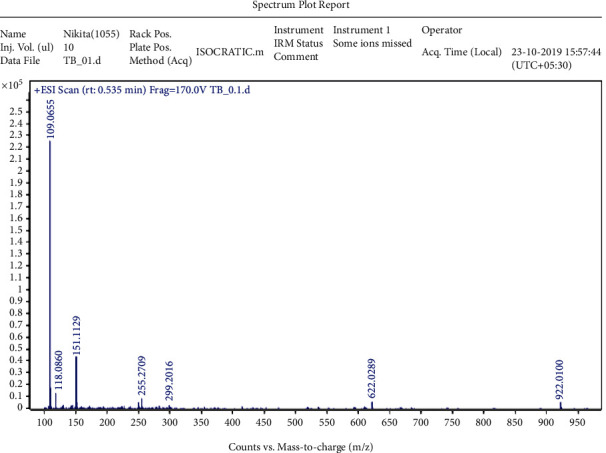
HRMS of the isolated compound.

**Figure 14:**
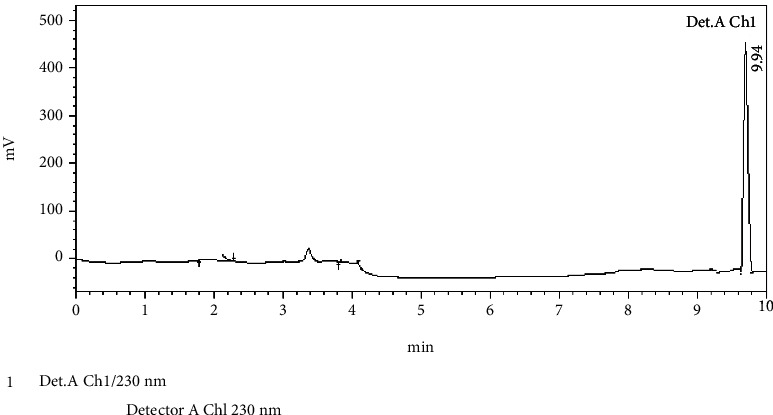
HPLC of the isolated compound.

Spectroscopic analysis has confirmed the presence of thymol (Figures 9–14). In the current study, the effects of thymol isolated from Trachyspermum ammi have been investigated for doses of 10 and 20 mg/kg on diabetes and diabetic neuropathy induced by streptozotocin in Wistar rats. The tests were also conducted for behaviour analysis, food, water consumption, lipid profile analysis, nociception, response latency, neuromuscular coordination, and histopathological analysis of the liver and sciatic nerve. A decrease in the blood glucose levels was noted by doses of 10 and 20 mg/kg of thymol. The reduction in blood glucose levels must be due to the stimulation of insulin secretions from beta cells that stimulate glucose metabolism and restore the remaining cells—this might be the best possible mechanism suggested by the standard drug glibenclamide [54].

This experiment has also shown protective effects of the isolated compound to treat diabetic neuropathy, which is a prominent effect of prolonged diabetes conditions. A loss of pain perception is noted in people who have diabetes due to extensive nerve damage; hence, there is the induction of peripheral neuropathy [55, 56].

The study revealed a delayed response in diabetic control group animals due to loss of perception and extensive damage to nerves, induced by prolonged diabetes. A gradual healing action was noted in treated groups which were most significant in groups receiving 20 mg/kg of thymol. In comparison, the diabetic groups have shown a trend of high sensitivity responses in nerves to delayed responses during week 12 due to extensive nerve damage. The treatment groups have also demonstrated a significant reduction in the paw withdrawal during thermal and mechanical hyperalgesia in the streptozotocin-induced Wistar rats which justifies the diabetic neuropathy protective action of thymol [57].

Loss of in nociception in diabetic animals must be due to the development of DN, which is the characterization of nerve degradation, leading to loss in the perception of pain [55, 58]. When an animal starts developing nerve damage, it tends to become lethargic and shows delayed responses due to progression of pain and development of diabetic neuropathy [59, 60]. The results obtained from the tail-flick test, thermal hyperalgesia, and cold hyperalgesia also reported a loss of perception of pain, hot or cold due to intense nerve damage in diabetic rats, and improved results in treated groups due to their nerve damage healing affect. The doses of 10 and 20 mg/kg have also shown improvement in the liver and sciatic nerve tissues, hence expressing the neuroprotection and antidiabetic action of isolated thymol.

In the analysis of vasa nervorum (longitudinal) in the sciatic nerves of Wistar rats on 12-week prominent signs of inflammation and damage, lipid degenerated axons showing focal peripheral axonal loss were noted in the diabetic group while the treated groups receiving 10 and 20 mg/kg thymol have shown minimal axonal degeneration which is a sign of healing and neuroprotective action of the drug. These healing signs are the recoveries of the damage induced by prolonged diabetes conditions. Similarly, the cells in the diabetic group have shown fatty necrosis and ballooning degradation, which represents disruptive cells in the liver. At the same time, the treated groups (10 and 20 mg/kg thymol) have shown normal hepatic cells with narrow sinusoids which are healthy liver cells. They signify the recovery of cells from diabetes. Hence, the sciatic nerve and liver cell histopathology hints on antidiabetic and neuroprotective thymol on Wistar rats.

Positive findings were recorded in thermal hyperalgesia, writhing, cold hyperalgesia responses, mechanical hyperalgesia, grip strength, spontaneous locomotor (exploratory) test, neuromuscular coordination tests, and histopathological and lipid profile analysis. The neuroprotective results were quite significant at the end of treatment that is by the end of 12 weeks. Uncontrollable lipid profiles have been the prime complications of diabetes mellitus, which is noted in 40% of the diabetes cases. Hypertriglyceridemia is a condition very commonly found in patients suffering from diabetes mellitus and is also seen in situations of insulin resistance either due to reduced catabolism or reduced production of lipoproteins [61].

Endothelial damages are caused due to the presence of superoxide and endogenous enzymes. An increase in aldose reductase and protein kinase C has shown connections with pain perceptions. Antioxidant protection is done by SOD which transforms the superoxide anions into H_2_O_2_. Endogenous enzymes SOD and MDA closely reciprocate to the oxidative stress. Elevation in the stress levels as depicted by MDA leads to lipid membrane disruption. This causes rearrangement of bonds in unsaturated fatty acids created by tissue damage. Increased levels of lipid peroxidase are linked to oxidative stress levels, which are decreased during the drug treatment process. Nitric oxide is an intracellular messenger, and it has a significant role in the pathological process as it combines with ROS (reactive oxygen species), an antioxidant. The process of oxidation affects cell molecules and is nonspecific [62, 63].

Superoxide anions lead to an increase in NO levels; hence, it forms peroxynitrite, which causes fast nitrosylation, DNA damage, cell death, and lipid peroxidation. These effects lead to untimely elevated pain and aggregation. Pyridyl porphyrin, the catalyst, leads to sensory neuropathy in the diabetic group. The results observed in the diabetic group are per previous studies showing worsening neuropathy [2, 64, 65].

Na^+^K^+^ATPase distribution must be uniform for proper generation and the conduction of bioelectricity. A reduction in chronic hyperglycemia caused a decrease in the Na^+^K^+^ATPase enzyme which caused inactivation of phosphate. This further leads to activation of polyol pathways. Na^+^K^+^ATPase enzyme is inhibited, but the restoration is preferred. In a recent study, thymol has shown to restore levels of Na^+^K^+^ATPase hence hinting its neuroprotective action [66].

Acceleration of the production of TNF-*α* in neural and microvascular tissues is a prominent feature of DN. This causes damage to nerves and microangiopathy in microvascular tissues. Hence, for treatment of diabetic neuropathy, a suppression in cytokine elevation is advised. Therefore, dose-dependency of the isolated compound thymol and its function in inhibition of cytokine levels is noted [67–69].

This study noted physiological, biochemical, and histopathological deviations in thymol-administered groups. The isolated compound has also shown efficient results when compared to a standard drug for restoring the levels of biomarkers, hence more effective in treating diabetic neuropathy. The critical factors responsible for neuropathic pain, oxidative stress, cytokine release, and tumor necrosis factor-*α* (TNF-*α*) were restored by thymol dosing in rats. These were also important in restoring the membrane bound inorganic phosphate activity and apoptotic conditions; henceforth, neuropathic pain mediation was regulated. Thymol aced the significant reduction of diabetic neuropathy pain by involving various mechanisms which lead to restoration of levels of Na^+^K^+^ATPase, inhibition of the elevated cytokines, reduction of TNF-*α*, and decrease in the NO levels. Thus, the antioxidant nature and healing action of thymol isolated from the herbal extract were signified and confirmed.

## 5. Conclusion

In conclusion, the treatment of thymol concentrations of 10 mg/kg and 20 mg/kg has ameliorated diabetes and conditions of diabetic neuropathy in STZ rats. Hence, it is obvious that thymol's oral dosing isolated from *Trachyspermum ammi* has improved the nociceptive latency, glucose levels, and lipid profiles and has subsequently shown healing effects in the liver and sciatic nerve of the treated groups where 20 mg/kg of thymol is more potent than its lower concentration group. The future aspects of this work suggest a detailed study on the mechanism of action to be performed to get better insights into thymol's role in the treatment of neuropathy, diabetes, and other metabolic disorders.

## Figures and Tables

**Table 1:** Experimental design.

S. No.	Group number	Group type	Dosing
1	Group I	Normal control	Normal control (2% *w*/*v* acacia)
2	Group II	Diabetic control	No drug
3	Group III	Standard	0.2 mg/kg of glibenclamide
4	Group IV	Treated 1	10 mg/kg of isolated compound
5	Group V	Treated 2	20 mg/kg of isolated compound.

**Table 2:** Fasting blood glucose testing during the antidiabetic study for 28 days.

S. No.	Treatment type	Fasting blood glucose level in mg/dl			
		Day 7	Day 14	Day 21	Day 28
1	Normal control	95.4 ± 0.18	96.35 ± 1.76	97.24 ± 2.04	97.03 ± 2.19
2	Diabetic control	157.21 ± 0.32^a^	261.18 ± 2.24^a^	297.12 ± 1.37^a^	308.27 ± 0.37^a^
3	Standard	154.01 ± 0.14^∗^	153.13 ± 1.91^∗^	141.32 ± 1.41^∗^	121.18 ± 3.23^∗^
4	Treated 1	152.14 ± 1.62^∗^	159.37 ± 0.37^∗^	149.38 ± 3.27^∗^	129.01 ± 3.45^∗^
5	Treated 2	1521.53 ± 1.61^∗^	152.16 ± 2.81^∗^	135.14 ± 2.47^∗^	117.12 ± 1.35^∗^

Values are expressed as the mean ± SEM; *n* = 6. One-way ANOVA; followed by Tukey-Kramer's multiple comparisons test: ^a^*P* < 0.05 in comparison with normal control and ^∗^*P* < 0.05 in comparison with the diabetic control.

**Table 3:** Record of the body weight postinduction of diabetes in Wistar rats on weeks 2, 4, 8, and 12.

S. No.	Treatment type	Body weight (g)			
		2^nd^ week	4^th^ week	8^th^ week	12^th^ week
1	Normal control	152.3 ± 2.03	168.27 ± 2.21	190.25 ± 5.13	231.45 ± 0.26
2	Diabetic control	154.10 ± 2.40^a^	149.15 ± 1.32^a^	144.27 ± 1.53^a^	132.36 ± 3.26^a^
3	Standard	153.13 ± 1.26	150.13 ± 0.37	159.23 ± 1.32^∗∗∗^	199.2 ± 1.12^∗∗∗^
4	Treated 1	155.24 ± 1.32	163.4 ± 2.24^∗∗∗^	168.25 ± 0.25^∗∗∗^	201.34 ± 1.38^∗∗∗^
5	Treated 2	159.24 ± 1.35	166.14 ± 2.14^∗∗∗^	182.17 ± 1.34^∗∗∗^	211.23 ± 1.42^∗∗∗^

Values are expressed as the mean ± SEM; *n* = 6. One-way ANOVA; Tukey-Kramer's multiple comparisons test: ^a^*P* < 0.001 in comparison with normal control; and ^∗∗∗^*P* < 0.001 in comparison with the diabetic control.

**Table 4:** Assessment of food and water consumption post treatment in Wistar rats.

S. No.	Treatment type	Average food & water consumption	
		Water intake (ml/day)	Food intake (g/day)
1	Normal control	13.46 ± 0.82	18.21 ± 1.34
2	Diabetic control	36.25 ± 6.34^a^	27.26 ± 0.31^a^
3	Standard	21.17 ± 2.35^∗∗∗^	21.14 ± 2.62^∗∗∗^
4	Treated 1	17.42 ± 1.35^∗∗∗^	22.47 ± 1.37^∗∗∗^
5	Treated 2	16.13 ± 0.59^∗∗∗^	21.02 ± 0.15^∗∗∗^

Values are expressed as the mean ± SEM; *n* = 6. One-way ANOVA; Tukey-Kramer's multiple comparisons test: ^a^*P* < 0.001 in comparison with normal control; and ^∗∗∗^*P* < 0.001 in comparison with the diabetic control.

**Table 5:** Effects on lipid profile of groups in Wistar rats.

S. No.	Treatment type	Lipid profile (mg/dl) posttreatment				
		TC	Triglyceride	LDL	HDL	VLDL
1	Normal control	74.29 ± 0.35	65.16 ± 0.28	36.29 ± 0.19	27.04 ± 4.15	13.28 ± 0.27
2	Diabetic control	143.29 ± 0.25^a^	157.21 ± 0.28^a^	94.19 ± 0.14^a^	15.19 ± 0.42^a^	46.38 ± 0.25^a^
3	Standard	77.23 ± 2.36^∗∗∗^	65.48 ± 1.49^∗∗∗^	37.16 ± 0.25^∗∗∗^	29.8 ± 2.46^∗∗∗^	15.12 ± 1.38^∗∗∗^
4	Treated 1	85.02 ± 1.91^∗∗∗^	72.26 ± 2.43^∗∗∗^	45.26 ± 2.36^∗∗∗^	20.35 ± 2.26^∗∗∗^	21.13 ± 1.27^∗∗∗^
5	Treated 2	74.59 ± 0.24^∗∗∗^	65.15 ± 1.28^∗∗∗^	37.24 ± 2.29^∗∗∗^	28.03 ± 0.29^∗∗∗^	14.01 ± 0.13^∗∗∗^

Values are expressed as the mean ± SEM; *n* = 6. One-way ANOVA; Tukey-Kramer's multiple comparisons test: ^a^*P* < 0.001 in comparison with normal control; and ^∗∗∗^*P* < 0.001 in comparison with the diabetic control.

**Table 6:** Thermal hyperalgesia: Eddy's hot plate test on animals in all groups to assess diabetic neuropathy in Wistar rats on weeks 2, 4, 8, and 12.

S. No.	Treatment type	Thermal hyperalgesia—Eddy's hot plate method—response time (s)			
		Reaction latency (2^nd^ week)	Reaction latency (4^th^ weeks)	Reaction latency (8^th^ weeks)	Reaction latency (12^th^ weeks)
1	Normal control	4.12 ± 1.43	4.19 ± 1.28	4.01 ± 1.03	4.01 ± 1.45
2	Diabetic control	6.77 ± 1.28^a^	5.19 ± 1.48^a^	8.23 ± 2.92^a^	9.85 ± 1.86^a^
3	Standard	6.25 ± 3.14	5.30 ± 4.19	5.89 ± 1.76^∗∗∗^	5.91 ± 1.74^∗∗∗^
4	Treated 1	6.92 ± 0.29	6.55 ± 1.32^∗∗∗^	5.18 ± 0.21^∗∗∗^	6.08 ± 1.41^∗∗∗^
5	Treated 2	6.01 ± 0.48	4.41 ± 1.34	5.11 ± 1.65^∗∗∗^	5.93 ± 0.24^∗∗∗^

Values are expressed as the mean ± SEM; *n* = 6. One-way ANOVA; followed by Tukey-Kramer's multiple comparisons test: ^a^*P* < 0.001 in comparison with normal control; ^∗∗∗^*P* < 0.001 in comparison with the diabetic control.

**Table 7:** Recoding the writhing responses to assess the neuropathic pain in all groups of Wistar rats on weeks 2, 4, 8, and 12.

S. No.	Treatment type	Number of writhing responses in ten minutes (counts)			
		(2^nd^ week)	(4^th^ weeks)	(8^th^ weeks)	(12^th^ weeks)
1	Normal control	10.01 ± 0.12	11.16 ± 0.22	10.24 ± 0.22	8.13 ± 1.32
2	Diabetic control	11.14 ± 1.44^a^	10.22 ± 6.13^a^	12.58 ± 4.80^a^	13.35 ± 0.35^a^
3	Standard	12.35 ± 2.24	9.15 ± 2.34	9.64 ± 2.32^∗∗∗^	8.94 ± 1.35^∗∗∗^
4	Treated 1	14.94 ± 4.25	9.24 ± 1.34	9.94 ± 1.31^∗∗∗^	8.28 ± 1.44^∗∗∗^
5	Treated 2	13.32 ± 1.32	9.14 ± 2.11	9.61 ± 0.21^∗∗∗^	8.24 ± 0.17^∗∗∗^

Values are expressed as the mean ± SEM; *n* = 6. One-way ANOVA; Tukey-Kramer's multiple comparisons test: ^a^*P* < 0.001 in comparison with normal control; and ^∗∗∗^*P* < 0.001 in comparison with the diabetic control.

**Table 8:** Cold hyperalgesia: acetone drop test in all groups in Wistar rats on weeks 2, 4, 8, and 12.

S. No.	Treatment type	Cold hyperalgesia (acetone drop test) in sec			
		Allodynia score in sec (2^nd^ week)	Allodynia score in sec (4^th^ weeks)	Allodynia score in sec (8^th^ weeks)	Allodynia score in sec (12^th^ weeks)
1	Normal control	4.62 ± 1.35	4.27 ± 0.25	4.07 ± 2.03	4.12 ± 1.38
2	Diabetic control	8.16 ± 2.18^a^	4.32 ± 1.91^a^	8.26 ± 0.54^a^	9.17 ± 1.34^a^
3	Standard	6.19 ± 0.37	5.19 ± 1.36	6.17 ± 1.11^∗∗∗^	6.11 ± 2.12^∗∗∗^
4	Treated 1	7.16 ± 1.15	5.85 ± 0.26^∗∗∗^	6.26 ± 2.46^∗∗∗^	5.93 ± 1.38^∗∗∗^
5	Treated 2	6.31 ± 0.35	5.16 ± 1.82	6.31 ± 1.81^∗∗∗^	5.15 ± 1.27^∗∗∗^

Values are expressed as the mean ± SEM; *n* = 6. One-way ANOVA; followed by Tukey-Kramer's multiple comparisons test: ^a^*P* < 0.001 in comparison with normal control; ^∗∗∗^*P* < 0.001 in comparison with the diabetic control.

**Table 9:** Mechanical hyperalgesia: pinprick test in all groups in Wistar rats on weeks 2, 4, 8, and 12.

S. No.	Treatment type	Mechanical hyperalgesia (pinprick test) sec			
		Response latency in sec (2^nd^ week)	Response latency in sec (4^th^ weeks)	Response latency in sec (8^th^ weeks)	Response late response latency in sec (12^th^ weeks)
1	Normal control	2.26 ± 1.36	2.19 ± 1.48	1.97 ± 1.39	2.18 ± 1.92
2	Diabetic control	5.16 ± 1.36^a^	4.96 ± 1.04^a^	6.71 ± 0.48^a^	9.71 ± 1.04^a^
3	Standard	4.22 ± 0.23^∗∗∗^	3.12 ± 2.05^∗∗∗^	4.43 ± 2.82^∗∗∗^	4.74 ± 2.72^∗∗∗^
4	Treated 1	5.01 ± 0.11	3.15 ± 0.45^∗∗∗^	4.21 ± 2.91^∗∗∗^	4.89 ± 1.25^∗∗∗^
5	Treated 2	4.14 ± 1.26^∗∗∗^	3.38 ± 1.26	4.96 ± 2.91^∗∗∗^	4.51 ± 0.21^∗∗∗^

Values are expressed as the mean ± SEM; *n* = 6. One-way ANOVA; Tukey-Kramer's multiple comparisons test: ^a^*P* < 0.001 in comparison with normal control; and ^∗∗∗^*P* < 0.001 in comparison with the diabetic control.

**Table 10:** Grip strength to record the response latency in sec in all groups in Wistar rats on weeks 2, 4, 8, and 12.

S. No.	Treatment type	Grip strength for each animal in a group ± SEM (*n* = 6) (sec)			
		Response latency in sec (2^th^ week)	Response latency in sec (4^th^ weeks)	Response latency in sec (8^th^ weeks)	Response latency in sec (12^th^ weeks)
1	Normal control	31.11 ± 0.13	30.97 ± 1.41	31.21 ± 1.91	34.31 ± 0.19
2	Diabetic control	13.03 ± 0.38^a^	8.93 ± 1.51^a^	6.49 ± 1.41^a^	4.01 ± 0.25^a^
3	Standard	15.14 ± 1.26^∗^	24.31 ± 0.87^∗^	25.56 ± 1.32^∗^	28.21 ± 2.13^∗^
4	Treated 1	15.77 ± 1.06^∗^	21.11 ± 3.71^∗^	23.27 ± 0.39^∗^	24.35 ± 1.16^∗^
5	Treated 2	19.03 ± 1.36^∗^	25.25 ± 0.27^∗^	26.18 ± 0.23^∗^	32.16 ± 1.62^∗^

Values are expressed as the mean ± SEM; *n* = 6. One-way ANOVA; followed by Tukey-Kramer's multiple comparisons test: ^a^*P* < 0.05 in comparison with normal control; ^∗∗∗^*P* < 0.05 in comparison with the diabetic control.

**Table 11:** Spontaneous locomotor (exploratory) test: actophotometer on all groups in Wistar rats on weeks 2, 4, 8, and 12.

S. No.	Treatment type	Actophotometer: counts per 5 minutes			
		Counts (2^nd^ week)	Counts (4^th^ weeks)	Counts (8^th^ weeks)	Counts (12^th^ weeks)
1	Normal control	121.36 ± 1.34	126.11 ± 2.52	122.52 ± 3.65	127.43 ± 2.01
2	Diabetic control	35.14 ± 3.26^a^	28.18 ± 0.14^a^	19.04 ± 1.81^a^	15.01 ± 3.24^a^
3	Standard	41.32 ± 2.42^∗^	70.27 ± 2.44^∗^	84.24 ± 2.22^∗^	93.25 ± 0.42^∗^
4	Treated 1	42.37 ± 3.25^∗^	68.26 ± 2.32^∗^	80.14 ± 3.62^∗^	90.33 ± 2.66^∗^
5	Treated 2	44.46 ± 3.23^∗^	72.24 ± 2.72^∗^	89.25 ± 2.43^∗^	100.24 ± 2.55^∗^

Values are expressed as the mean ± SEM; *n* = 6. One-way ANOVA; followed by Tukey-Kramer's multiple comparisons test: ^a^*P* < 0.05 in comparison with normal control; ∗P <0.05 in comparison with the diabetic control.

**Table 12:** Neuromuscular coordination test (motor coordination): rotarod test on all groups in Wistar rats on weeks 0, 2, 4, and 6.

S. No.	Treatment type	Rotarod test (counts)			
		Counts (2^nd^ week)	Counts (4^th^ weeks)	Counts (8^th^ weeks)	Counts (12^th^ weeks)
1	Normal control	123.14 ± 2.01	125.1 ± 0.25	127.41 ± 1.02	129.37 ± 4.14
2	Diabetic control	43.11 ± 1.10^a^	21.41 ± 3.14^a^	16.24 ± 0.61^a^	10.39 ± 2.05^a^
3	Standard	50.26 ± 4.39	84.01 ± 5.23^∗∗∗^	97.52 ± 2.19^∗∗∗^	109.13 ± 0.28^∗∗∗^
4	Treated 1	49.40 ± 2.91^∗∗∗^	78.27 ± 2.71^∗∗∗^	89.24 ± 3.27^∗∗∗^	100.25 ± 4.82^∗∗∗^
5	Treated 2	52.15 ± 4.16	96.13 ± 3.37^∗∗∗^	99.24 ± 4.46^∗∗∗^	102.18 ± 0.31^∗∗∗^

Values are expressed as the mean ± SEM; *n* = 6. One-way ANOVA; Tukey-Kramer's multiple comparisons test: ^a^*P* < 0.001 in comparison with normal control; ^∗∗∗^*P* < 0.001 in comparison with the diabetic control.

**Table 13:** Effect of thymol on endogenous biomarkers in rats.

S. No.	Treatment type	Endogenous biomarkers				
	Group type	SOD (U/mg of protein)	NO (*μ*g/ml)	LPO (nM/mg of protein)	Na^+^K^+^ATPase (*μ*mol/mg of protein)	TNF-*α* (pg/ml)
1	Normal control	24.03 ± 1.42	104.03 ± 1.36	2.34 ± 1.13	10.11 ± 1.23	51.35 ± 1.13
2	Diabetic control	5.32 ± 1.13^a^	303.11 ± 2.11^a^	9.11 ± 1.13^a^	2.34 ± 0.17^a^	160.01 ± 1.21^a^
3	Standard	16.12 ± 0.12^∗^	224.32 ± 2.12^∗^	6.13 ± 1.41^∗^	6.21 ± 0.21^∗^	119.13 ± 1.12^∗^
4	Treated 1	21.11 ± 1.23^∗^	158.09 ± 2.12^∗^	4.13 ± 1.23^∗^	7.31 ± 2.03^∗^	90.21 ± 1.13^∗^
5	Treated 2	23.24 ± 1.22^∗^	129.22 ± 2.01^∗^	2.71 ± 1.31^∗^	9.34 ± 1.34^∗^	67.25 ± 0.21^∗^

Values are expressed as the mean ± SEM; *n* = 6. One-way ANOVA; Tukey-Kramer's multiple comparisons test: ^a^*P* < 0.05 compared to normal control; ^⁎^*P* < 0.05 in comparison with the diabetic control.

## Data Availability

Data will be available on request to the corresponding author.

## Abbreviations

DPN: Diabetic polyneuropathy
DN: Diabetic neuropathy
CPCSEA: Committee for the Purpose of Control and Supervision of Experiments on Animals
DMSO: Dimethyl sulfoxide
OECD: Organisation for Economic Co-operation and Development
DNA: Deoxyribonucleic acid
DM: Diabetes mellitus
TMB: 3,3′,5,5′-Tetramethylbenzidine
UV spectroscopy: Ultraviolet spectroscopy
TLC: Thin Layer Chromatography
IAEC: Institute Animal Ethics Committee
FTIR: Fourier-transform infrared spectroscopy
HPLC: High-Performance Liquid Chromatography
HRMS: High-resolution mass spectrometry
NMR: Nuclear Magnetic Resonance
STZ: Streptozotocin
SOD: Superoxide dismutase
MDA: Malondialdehyde
NO: Nitric oxide
LPO: Neutral Lipid Peroxidase
Na^+^K^+^ATPase: Membrane-bound inorganic phosphate
TNF-*α*: Tumor necrosis factor alpha
Pyridyl porphyrin: Fe(III)tetrakis-2-(N-triethylene glycol monomethyl ether.
